# A Polyuridine Insertion in the 3′ Untranslated Region of Classical Swine Fever Virus Activates Immunity and Reduces Viral Virulence in Piglets

**DOI:** 10.1128/JVI.01214-19

**Published:** 2020-01-06

**Authors:** Miaomiao Wang, Matthias Liniger, Sara Muñoz-González, José Alejandro Bohórquez, Yoandry Hinojosa, Markus Gerber, Sergio López-Soria, Rosa Rosell, Nicolas Ruggli, Llilianne Ganges

**Affiliations:** a OIE Reference Laboratory for classical swine fever, IRTA-CReSA, Barcelona, Spain; b Institute of Virology and Immunology, Mittelhäusern, Switzerland; c Department of Infectious Diseases and Pathobiology, University of Bern, Bern, Switzerland; d Graduate School for Cellular and Biomedical Sciences, University of Bern, Bern, Switzerland; e Centro Nacional de Sanidad Agropecuaria, Mayabeque, Cuba; f Departament d’Agricultura, Ramadería, Pesca, Alimentació I Medi Natural i Rural, Generalitat de Catalunya, Barcelona, Spain; Loyola University Chicago

**Keywords:** 3′ UTR, CSFV, polyuridine insertion, viral replication, virulence

## Abstract

Low-virulence classical swine fever virus (CSFV) strains make CSF eradication particularly difficult. Few data are available on the molecular determinants of CSFV virulence. The aim of the present study was to assess a possible role for CSFV virulence of a unique, uninterrupted 36-uridine (poly-U) sequence found in the 3′ untranslated region (3′ UTR) of the low-virulence CSFV isolate Pinar de Rio (PdR). To this end, a pair of cDNA-derived viruses based on the PdR backbone were generated, one carrying the long poly-U insertion in the 3′ UTR (vPdR-36U) and the other harboring the standard 5 uridines at this position (vPdR-5U). Two groups of 20 5-day-old piglets were infected with vPdR-36U and vPdR-5U. Ten contact piglets were added to each group. Disease progression, virus replication, and immune responses were monitored for 5 weeks. The vPdR-5U virus was significantly more virulent than the vPdR-36U virus, with more severe disease, higher mortality, and significantly higher viral loads in serum and body secretions, despite similar replication characteristics in cell culture. The two viruses were transmitted to all contact piglets. Ninety percent of the piglets infected with vPdR-36U seroconverted, while only one vPdR-5U-infected piglet developed antibodies. The vPdR-5U-infected piglets showed only transient alpha interferon (IFN-α) responses in serum after 1 week of infection, while the vPdR-36U-infected piglets showed sustained IFN-α levels during the first 2 weeks. Taken together, these data show that the 3′ UTR poly-U insertion acquired by the PdR isolate reduces viral virulence and activates the innate and humoral immune responses without affecting viral transmission.

**IMPORTANCE** Classical swine fever (CSF), a highly contagious viral disease of pigs, is still endemic in some countries of Asia and Central and South America. Considering that the 3′ untranslated region (3′ UTR) plays an important role in flavivirus replication, the present study showed for the first time that a long polyuridine sequence acquired in the 3′ UTR by an endemic CSFV isolate can activate immunity, control viral replication, and modulate disease in piglets. Our findings provide new avenues for the development of novel vaccines against infections with CSF virus and other flaviviruses. Knowledge of molecular virulence determinants is also relevant for future development of rapid and efficient diagnostic tools for the prediction of the virulence of field isolates and for efficient CSF control.

## Abstract (precis)

Classical swine fever (CSF), a highly contagious viral disease of pigs, is still endemic in some countries of Asia and Central and South America. Considering that the 3′ untranslated region (3′ UTR) plays an important role in flavivirus replication, the present study showed for the first time that a long polyuridine sequence acquired in the 3′ UTR by an endemic CSFV isolate can activate immunity, control viral replication, and modulate disease in piglets. Our findings provide new avenues for the development of novel vaccines against infections with CSF virus and other flaviviruses. Knowledge of molecular virulence determinants is also relevant for future development of rapid and efficient diagnostic tools for the prediction of the virulence of field isolates and for efficient CSF control.

## INTRODUCTION

Classical swine fever (CSF) is a highly contagious viral disease that affects domestic and wild pigs. Due to its socioeconomic importance in domestic pigs, CSF is notifiable to the World Organisation for Animal Health (OIE). The disease is characterized by a broad spectrum of clinical signs. The individual outcome depends on several factors, such as the age of the infected animal and the virulence of the virus (1). Currently, CSF is endemic in several countries of South and Central America and some parts of Asia (2).

The disease is caused by CSF virus (CSFV), which belongs to the *Pestivirus* genus within the *Flaviviridae* family (3). CSFV is composed of a lipid envelope, a capsid, and a single-stranded, positive-sense RNA genome of 12.3 kb. The genome carries a single long open reading frame (ORF) flanked by 5′ and 3′ untranslated regions (UTR) that are important for genome replication and initiation of viral protein translation. The ORF encodes four structural and eight nonstructural proteins (4). The envelope glycoprotein E2 represents the major immunogenic protein of pestiviruses, plays a central role in virus entry, and is associated with virulence (5). CSFV strains can be classified as of high, moderate, or low virulence. While infections with highly virulent strains induce acute disease, strains with a lower degree of virulence can result in chronic, persistent, and subclinical forms of the disease (6, 7). For these reasons, low-virulence CSFV strains are particularly challenging for CSF eradication. Little is known on the molecular determinants of CSFV attenuation and of disease pathogenesis. Recently, a unique uninterrupted polyuridine (poly-U) sequence of an average length of 36 nucleotides was found in the 3′ UTR of the low-virulence CSFV strain Pinar del Rio (PdR), isolated from a country of endemicity under a vaccination program (8, 9). According to GenBank, all other CSFV genomes known to date harbor 4 or 5 uridines at this position of the 3′ UTR (9). The highly virulent CSFV strain Margarita, likely the parental strain of the PdR virus (9), also carries the standard 5 uridines at this position. However, a similar 6- to 32-nucleotide-long uridine-rich insertion was found in the 3′ UTR of several attenuated CSF vaccine virus strains, but this sequence was located approximately 90 nucleotides upstream of the PdR poly-U insertion (10, 11). Previous studies showed that the 3′ UTR of flaviviruses are implicated in virus replication (12, 13). Accordingly, it was speculated that the uridine-rich sequences of these vaccine strains and the poly-U insertion of PdR may contribute to viral attenuation (9, 10).

In the present study, we investigated the potential role of the poly-U sequence of the PdR strain in attenuation and disease pathogenesis. To this end, we generated two functional cDNA clones, one with the sequence of the original PdR isolate carrying the 36-uridine sequence (pPdR-36U) and a mutant with the standard 5 uridines (pPdR-5U) at this position. The viruses were rescued from the respective cDNA clones and used to assess the role of the poly-U sequence for replication in cell culture and for virulence and transmission in newborn piglets.

## RESULTS

### CSFV PdR recombinants with 36 and 5 uridines in the 3′ UTR do not differ in replication in cell culture.

**FIG 1:**
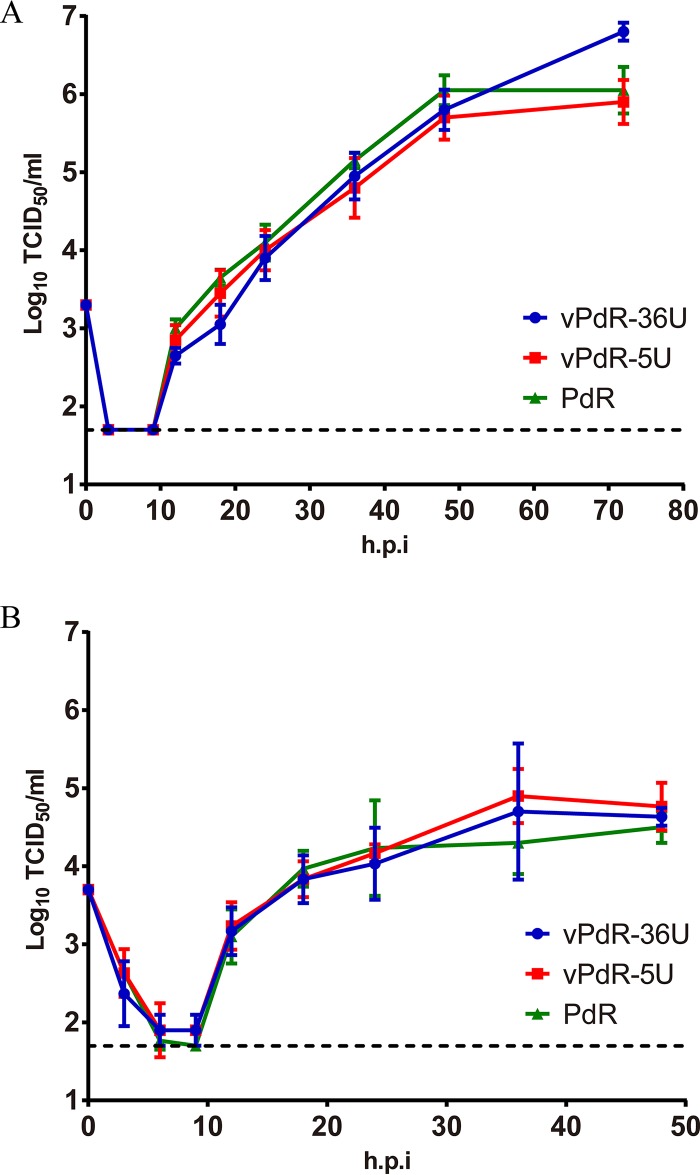
Virus replication kinetics in PEDSV.15 cells and porcine macrophages. PEDSV.15 cells (A) and porcine monocyte-derived macrophages (B) were infected in quadruplicate and in triplicate, respectively, with CSFV PdR and with cDNA-derived vPdR-36U and vPdR-5U at an MOI of 0.02 TCID_50_/cell based on titers obtained in the homologous cell type (plotted on day 0). At the indicated hours postinfection (h.p.i), virus was harvested by one freeze-thaw cycle and the infectious titer was determined in PEDSV.15 (A) or in SK-6 cells (B) by endpoint dilution. The limit of detection of the titration (1.7 log_10_ TCID_50_/ml) is represented with a dashed line. Each point represents the mean titer from four parallel infections in panel A and three in panel B, with error bars showing the SDs.

In order to study the role of the poly-U sequence found in the 3′ UTR of CSFV PdR, a functional cDNA clone of the PdR strain (pPdR-36U) and a mutant with the standard 5 uridines at this position (pPdR-5U) were constructed as described in Materials and Methods. The vPdR-36U and vPdR-5U viruses were rescued from the respective full-length cDNA clones by transfection of the porcine aortic endothelial cell line PEDSV.15 with *in vitro*-transcribed RNA (see Materials and Methods). For the two constructs, the specific infectivity of the RNA transcripts and the virus titer in PEDSV.15 cells 65 h after transfection were higher than 10^5^ focus-forming units/μg of RNA and 5 × 10^6^ 50% tissue culture infective doses (TCID_50_)/ml, respectively, which demonstrates the functionality of the two cDNA clones. After one additional passage in PEDSV.15 cells, the complete nucleotide sequence of the two viruses was determined, which confirmed the presence of the 36- and 5-uridine sequences in the 3′ UTR of the respective viruses and excluded any other difference or accidental mutation. Thus, vPdR-36U and the parental PdR virus (GenBank accession number KX576461) have identical consensus sequences. The growth characteristics of vPdR-36U, vPdR-5U, and the PdR isolate were analyzed in PEDSV.15 cells and in porcine monocyte-derived macrophages. The parental and the two cDNA-derived viruses did not differ in their replication kinetics (Fig. 1). This validates vPdR-36U as a functional cDNA-derived PdR strain. It demonstrates also that the poly-U sequence does not influence the basic replication characteristics of the virus in cell culture.

### The 36-uridine sequence in the 3′ UTR of PdR is associated with reduced disease severity in piglets.

**FIG 2:**
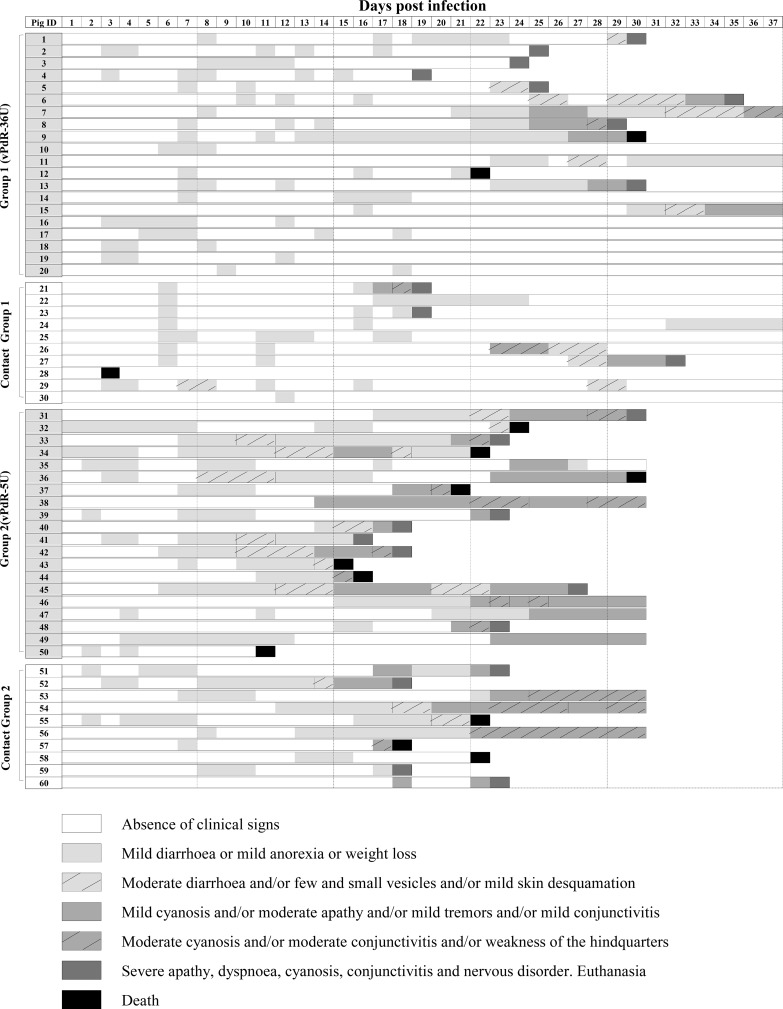
Individual clinical signs monitored after CSFV infection. Animals 1 to 20 (group 1) were infected with vPdR-36U and animals 21 to 30 were added to group 1 as contact animals after 24 h. Animals 31 to 50 were infected with vPdR-5U (group 2) and animals 51 to 60 were added to group 2 as contact animals after 24 h. The piglets were monitored clinically on a daily basis during the 37 days of the study. The severity of the clinical signs is represented by different shades of gray with or without hatches as shown in the key.

In order to determine whether the long poly-U sequence of the PdR virus has an effect on virulence *in vivo*, two groups of 5-day-old piglets (20 piglets each) were inoculated with vPdR-36U (group 1) and vPdR-5U (group 2). Notably, the swine is the natural host for CSFV, and piglets are highly susceptible to infection. Virus transmission was assessed by addition of 10 naive piglets to each group 24 h later (contact groups). The clinical signs were monitored daily for each individual animal in a blinded manner (Fig. 2), and a clinical score value was assigned accordingly (Data S1).

**FIG 3:**
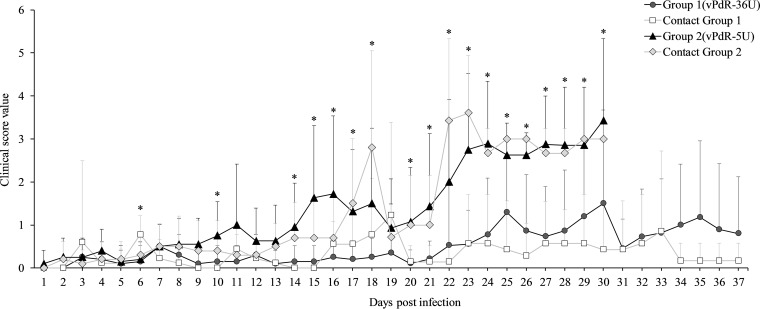
Mean clinical score values. The mean daily clinical score value (from zero to six) was calculated for group 1 (vPdR-36U-inoculated) and group 2 (vPdR-5U-inoculated) and for their respective contact groups. Asterisks indicate statistically significant differences of the mean clinical scores between the groups (*P* < 0.05).

During the first week after infection, very mild diarrhea and apathy were observed with both viruses, resulting in mean clinical scores close to zero (Fig. 3), with the exception of one of the vPdR-36U contact animals that died on the third day of the trial (Fig. 2 and Data S1). No significant difference was found between the groups during that time, except for the vPdR-36U contact animals compared with the other groups at 6 days postinfection (dpi) (*P* = 0.046) (Fig. 3).

Significant differences in disease manifestation between the groups appeared in the second week of infection. During this time, only mild sporadic diarrhea was observed in vPdR-36U-infected piglets, including the contact animals, which had clinical scores between 0 and 1. Meanwhile, the majority of vPdR-5U-infected piglets (inoculated and contacts) developed diarrhea, with some of them also showing other clinical signs, resulting in clinical scores between 1 and 2 (Fig. 2 and Data S1). One animal from group 2 died suddenly at 11 dpi (piglet 50 [Fig. 2]). Accordingly, during the second week, the vPdR-5U-infected piglets showed significantly higher clinical scores than the vPdR-36U-infected piglets at 10 and 14 dpi (*P* = 0.047 and 0.018, respectively) (Fig. 3).

During the third week postinfection, the disease was consistently more severe in vPdR-5U- than in vPdR-36U-infected piglets, with significantly higher clinical scores between the two groups on 5 out of 7 days (*P* ≤ 0.042) (Fig. 3). Specifically, during this week, nearly all vPdR-36U-inoculated and remaining contact piglets were either clinically healthy or developed only mild sporadic diarrhea (clinical scores between 0 and 1). Nevertheless, one vPdR-36U-inoculated animal (number 4) and two contact animals had to be euthanized at 19 dpi due to high clinical score (Fig. 2 and Data S1). At the same time, most of the surviving vPdR-5U-inoculated animals developed clinical signs with scores of >3. Three piglets had to be euthanized and three others died suddenly (Fig. 2 and Data S1). Similarly, in the vPdR-5U contact group, the piglets developed progressive clinical signs, most of them with scores of >3; one died, and two had to be euthanized at 18 dpi (Fig. 2 and Data S1).

From the fourth week on, 7 of the 19 remaining vPdR-36U-infected piglets (group 1) were healthy, while 2 died suddenly. Eventually, seven piglets of this group were euthanized before the end of the experiment with clinical scores of 5 (Fig. 2). Among the contact animals of this group, five were clinically healthy until the end of the study and the remaining two showed clinical signs; one of them recovered during the fifth week, whereas the other had to be euthanized due to high clinical score (Fig. 2). In contrast, at the same time, all but one of the remaining vPdR-5U-inoculated and contact animals were severely ill. Considering that, the experiment was terminated by euthanasia of all remaining animals at 30 dpi after some animals had reached the endpoint criteria or were found dead (Fig. 2 and Data S1). Overall, from 22 dpi until 30 dpi, significantly higher clinical scores were observed in vPdR-5U-infected piglets than in vPdR-36U-infected piglets (*P* ≤ 0.032), especially at 23 dpi, with *P* < 0.001 (Fig. 3).

### The long poly-U sequence of PdR is associated with a prolonged IFN-α response.

**FIG 4:**
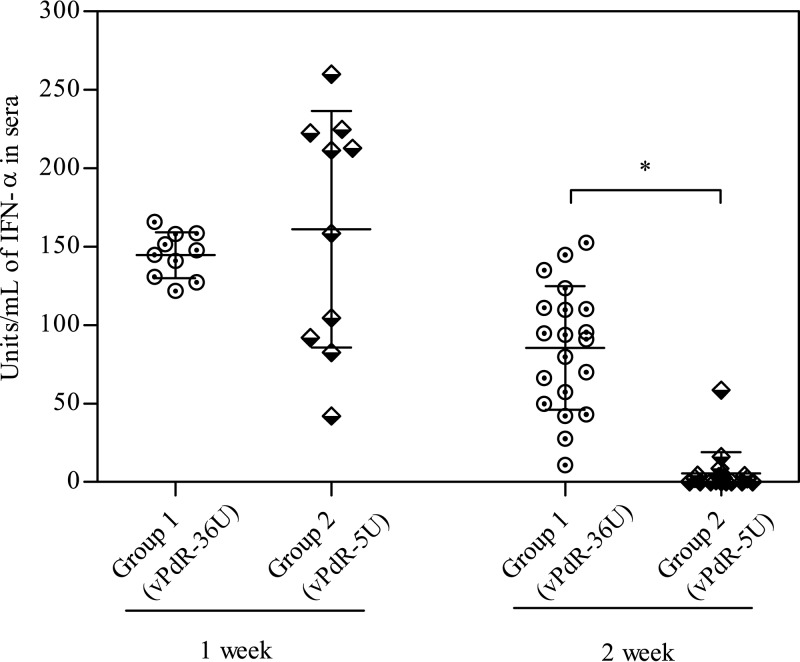
IFN-α levels in sera of CSFV-infected animals during the first 2 weeks postinfection. IFN-α levels were determined at 1 and 2 weeks postinfection in group 1 (vPdR-36U inoculated) and group 2 (vPdR-5U inoculated). The asterisk indicates a statistically significant difference between group 1 and group 2 at 2 weeks postinfection (*P* < 0.001).

No significant differences in the mean IFN-α levels were detected in the sera of the two groups after 1 week of infection; however, the IFN-α responses were more variable among the vPdR-5U-infected piglets (Fig. 4). Interestingly, a statistically significant difference between the IFN-α values of the two groups was observed in the second week postinfection (*P* < 0.001). All vPdR-36U-infected animals had detectable serum IFN-α levels at this time, while IFN-α was undetectable in most of the animals infected with vPdR-5U (Fig. 4).

### The poly-U insertion is related to lower viral replication *in vivo*.

**FIG 5:**
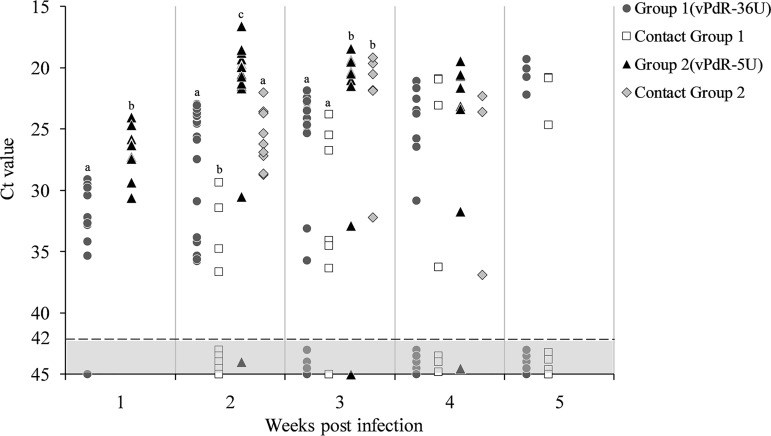
Detection of CSFV RNA in sera at weekly intervals. The sera were analyzed by RT-qPCR for the CSFV RNA content at weekly intervals during the whole experiment. *C_T_* values above 42 (light gray area with dotted line) were considered negative. For each week, the same letters represent no significant differences and different letters represent statistically significant differences between groups (*P* < 0.001).

During the first 3 weeks postinfection, the cycle threshold (*C_T_*) values for viral RNA in the sera of the piglets inoculated with vPdR-36U were significantly higher than the *C_T_* values measured in the sera of the vPdR-5U-inoculated piglets (*P* < 0.001) (Fig. 5). The same was true for the corresponding contact animals during the second and third weeks postinfection (Fig. 5). At these times, the contact piglets of the vPdR-36U-infected animals had the lowest viral RNA loads overall. Importantly, the highest viral RNA levels in the serum (*C_T_* values below 23) were found at 2 and 3 weeks postinfection in most of the vPdR-5U-inoculated piglets (group 2) and in their contact animals on week 3; these values were significantly higher (*P* < 0.001) than the levels found in vPdR-36U-inoculated (group 1) and contact animals (Fig. 5). At 4 weeks postinfection, 38% and 43% of the vPdR-36U-inoculated and contact piglets, respectively, had cleared the virus, i.e., were negative for CSFV RNA by quantitative reverse transcription-PCR (RT-qPCR), while 15 and 28%, respectively, had still high viral RNA levels. In contrast, only 12% of the vPdR-5U-inoculated piglets and none of their contact animals had cleared the virus (Fig. 5), with still high CSFV RNA levels in 50% and 33% of the surviving piglets, respectively. At 5 weeks after infection, the proportion of CSFV RNA-negative sera increased with respect to the previous week in the groups of vPdR-36U-inoculated and contact piglets, reaching 55 and 57%, respectively (Fig. 5). As mentioned above, none of the vPdR-5U-inoculated and contact animals survived at this time of the trial.

In terms of virus isolation at 3 weeks postinfection, virus was recovered from all sera that were positive by RT-qPCR with values below 32.22 (Table 1). Interestingly, virus was isolated from serum of 11 out of the 13 (85%) surviving vPdR-5U-inoculated piglets and from all their remaining contact animals. This contrasted with the vPdR-36U-inoculated piglets and their contact animals, for which virus could be recovered from only 12 out of 18 (66%) and 3 out of 7 (43%) remaining animals, respectively (Table 1). Finally, the titers of the virus-positive sera were in agreement with the RT-qPCR data (Table 1). From this we conclude that the modified PdR virus with 5 instead of 36 uridines in the 3′ UTR replicates more efficiently in piglets than the vPdR-36U virus.

**TABLE 1:** CSFV isolation and titration in the serum samples at 3 weeks postinfection^*a*^

Pig group	Pig no.	*C_T_* value	CSFV isolation	Titer^*b*^	Titer^*b*^	CSFV isolation	*C_T_* value	Pig no.	Pig group
Group 1 (vPdR-36U)	1	24.49	+	10^4.1^	10^5.5^	+	20.40	31	Group 2 (vPdR-5U)
	2	22.45	+	10^4.5^	10^5.4^	+	20.99	32	
	3	25.23	+	10^3.7^	10^6^	+	19.54	33	
	4	†			10^6.7^	+	18.41	34	
	5	23.40	+	10^4.6^		−	Undet.	35	
	6	24.11	+	10^4.2^		−	32.90	36	
	7	22.81	+	10^4.5^			†	37	
	8	23.51	+	10^4.6^	10^5.6^	+	20.34	38	
	9	25.33	+	10^3.5^	10^5.3^	+	20.47	39	
	10	Undet.	−				†	40	
	11	22.73	+	10^4.5^			†	41	
	12	†					†	42	
	13	24.56	+	10^4.1^			†	43	
	14	Undet.	−				†	44	
	15	33.13	−		10 ^6^	+	19.41	45	
	16	24.68	+	10^4.1^	10^6.2^	+	19.37	46	
	17	35.72	−		10^6.1^	+	19.33	47	
	18	Undet.	−		10^5.8^	+	19.50	48	
	19	21.85	+	10^5.3^	10^5.4^	+	21.48	49	
	20	Undet.	−				†	50	
Contact group 1	21	†			10^6^	+	19.64	51	Contact group 2
	22	34.1	−				†	52	
	23	†			10^1^	+	32.22	53	
	24	25.50	+	10^3.7^	10^5.2^	+	21.82	54	
	25	Undet.	−		10^6.8^	+	19.14	55	
	26	34.51	−		10^5.4^	+	21.84	56	
	27	26.76	+	10^3.5^			†	57	
	28	†					†	58	
	29	23.82	+	10^4.8^			†	59	
	30	36.35	−		10^5.5^	+	20.51	60	

a †, the animal died or was euthanized before 3 weeks postinfection; +, the sera were positive by CSFV isolation; –, the sera were negative by CSFV isolation. Undet., undetermined.

b Virus titration in TCID_50_ per milliliter.

### The long poly-U sequence is associated with reduced viral shedding from infected animals.

**FIG 6:**
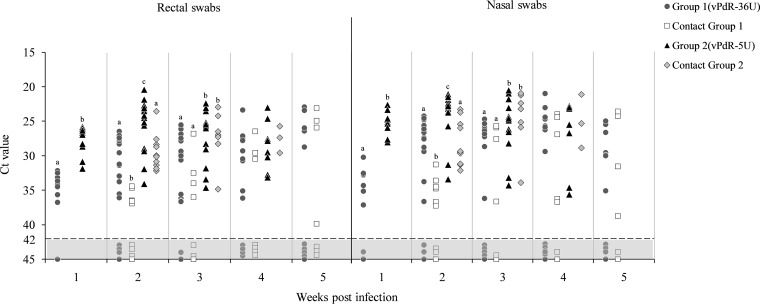
Detection of CSFV RNA in rectal and nasal swabs at weekly intervals. The CSFV RNA present in nasal and rectal swabs was evaluated weekly during the whole experiment. *C_T_* values above 42 (light gray area with dotted line) were considered negative. For each week, the same letters represent no significant differences and different letters represent significant differences between groups (*P* < 0.001).

All the rectal and nasal swabs from the vPdR-5U-infected piglets (group 2), including the contact animals, were positive for viral RNA by RT-qPCR throughout the study, unlike for the vPdR-36U-infected piglets (group 1 and contact group 1), for which a considerable number of negative samples were detected (Fig. 6). The differences in viral RNA levels in rectal and nasal swabs between the vPdR-5U- and vPdR-36U-infected piglets were in perfect agreement with the RT-qPCR data obtained from serum (see above and Fig. 5). During the first 3 weeks postinfection, the vPdR-5U-infected animals showed significantly lower *C_T_* values, corresponding to higher CSFV RNA loads, than the vPdR-36U-infected animals (*P* < 0.001) (Fig. 6). At 4 weeks postinfection, the rectal and nasal swabs of all the surviving vPdR-5U-infected animals—including contacts—remained RT-qPCR positive, while they were negative for 4 out of 14 and 2 out of 7 vPdR-36U-inoculated and contact piglets, respectively. In the fifth week after infection, only the vPdR-36U-infected piglets survived, showing similar viral RNA profiles in the nasal and rectal swabs (Fig. 6).

### The poly-U sequence is associated with stronger antibody responses to infection.

**FIG 7:**
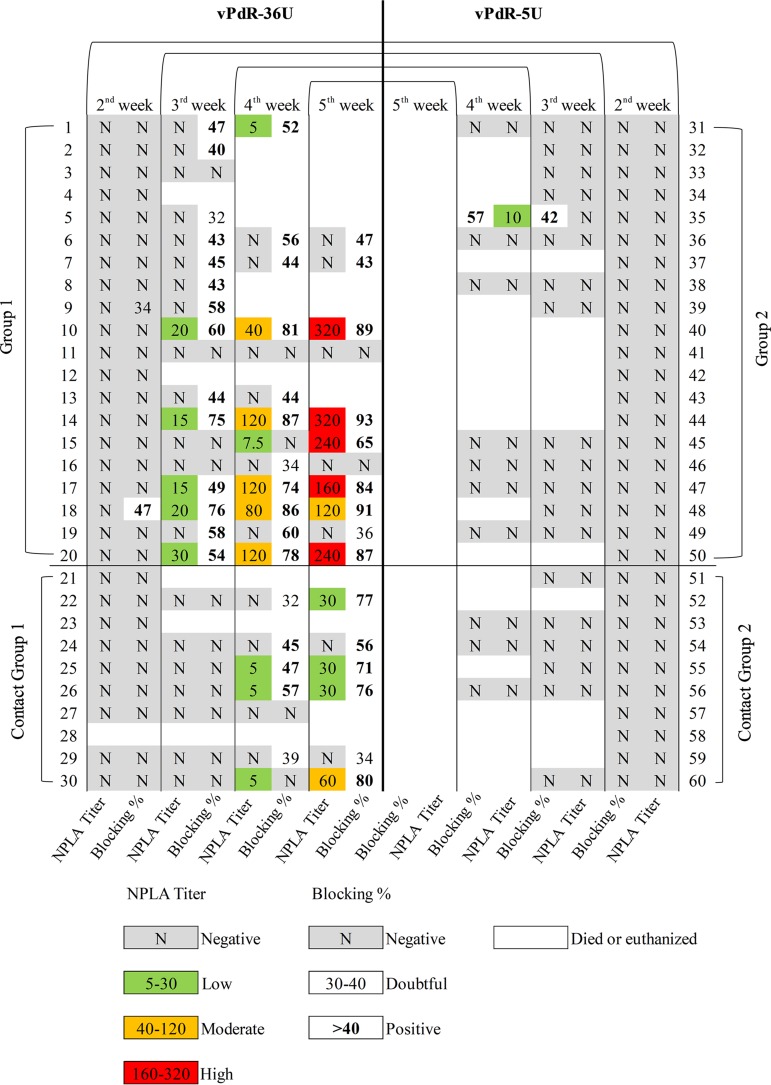
Assessment of the humoral immune responses after vPdR-36U versus vPdR-5U infection at weekly intervals. The antibody response against the E2 glycoprotein was detected by ELISA and is represented as blocking percentage. The neutralizing antibody titers after infection were evaluated by the NPLA.

Interestingly, there was a striking difference in terms of antibody responses between the vPdR-36U-infected (group 1) and vPdR-5U-infected (group 2) piglets (Fig. 7). One of the vPdR-36U-infected animals (number 18) seroconverted against E2 during the second week postinfection, and by the third week 13 out of 18 piglets (72%) had developed E2-specific antibody responses as detected by a blocking enzyme-linked immunosorbent assay (ELISA). At 4 weeks after infection, 10 out of 13 surviving piglets from this group were positive by ELISA, while 2 piglets remained negative until the end. The vPdR-36U contact piglets started to seroconvert during the 4th week, with 3 positive animals out of 7. One week later, 5 out of the 6 remaining contact piglets were seropositive. In contrast, only one of the vPdR-5U-inoculated piglets responded with E2-specific antibodies at 3 and 4 weeks postinfection, and all contact piglets remained seronegative throughout the trial (Fig. 7).

Accordingly, all the piglets were negative for neutralizing antibodies during the first 2 weeks of the experiment as measured with the neutralization peroxidase‐linked assay (NPLA). By the third week postinfection, 5 out of 18 vPdR-36U-inoculated animals had developed neutralizing antibodies, with titers between 1:15 and 1:30. At 4 weeks after infection, seven vPdR-36U-inoculated and three contact piglets were positive in the NPLA, with titers up to 1:120 and 1:5, respectively. The neutralizing antibody responses increased during the fifth week postinfection, with titers of 1:120 to 1:320 in 6 out of 11 surviving vPdR-36U-infected piglets and of 1:30 to 1:60 in 4 out of the 6 remaining contact animals (Fig. 7). For the vPdR-5U-infected piglets, the single animal that had seroconverted had also a 1:10 neutralizing antibody titer 1 week later.

### The poly-U sequence in vPdR-36U was conserved between inoculated and contact animals, despite not being completely stable.

Conventional RT-PCR and nucleotide sequencing were used to amplify and analyze the CSFV 3′ UTR from the sera of all inoculated and contact piglets 3 weeks postinfection. RT-PCR fragments were obtained only with the samples that had *C_T_* values below 32.9. Consequently, there was a smaller proportion of RT-PCR-positive samples with vPdR-36U- than with vPdR-5U-infected piglets (Table 1). From vPdR-36U-inoculated piglets, two fragments were obtained by RT-PCR in 5 out of 12 positive samples. After sequencing, these fragments showed two profiles, one with 36 uridines and the other with 4 or 5 uridines (data not shown). For the corresponding contact animals, one out of three RT-PCR positive samples showed one specific fragment with the 36 uridines while the other two samples revealed two fragments with 36 and 5 uridines. Finally, the 3′ UTR amplified from the sera of vPdR-5U-inoculated and contact animals showed the same fragment in all the samples analyzed with the expected 5 uridines (data not shown).

## DISCUSSION

Long-term prevalence and evolution of CSFV in regions of endemicity have resulted in viruses with a broad range of clinical manifestations, from severe to mild disease (14, 15). In this context, several studies have emphasized the relevance of moderate- and low-virulence CSFV strains for virus persistence in countries of endemicity under vaccination programs, representing a challenge for CSFV eradication (2, 9). Despite this, little is known on the genetic changes that are selected in the CSFV genomes in an endemic situation, leading to virus attenuation and persistence in the field. Therefore, the present study tackled these aspects by building on the PdR field isolate that resulted from natural CSFV evolution under endemic conditions for over 3 decades (9, 14). During circulation in the field, this strain has acquired a unique uninterrupted poly-U sequence of an average length of 36 nucleotides in the 3′ UTR that is absent in the related highly virulent Margarita virus (9). It should be noted that the Margarita strain was proposed to be the most probable origin of the Cuban outbreak in the 1990s and also is likely the parental strain for the PdR isolate (16, 17).

We used reverse genetics to characterize the role of the poly-U sequence for virulence and transmission in piglets by comparing cDNA-derived PdR with 36 and 5 uridines in the 3′ UTR, i.e., vPdR-36U and vPdR-5U, respectively. In accordance with previous data reported for the CSFV PdR strain isolated from the field (14), the clinical signs observed after infection with the vPdR-36U virus were mild and unspecific in newborn piglets (7). In contrast, piglets infected with vPdR-5U virus showed severe clinical signs, ranging from diarrhea, cyanosis, and hemorrhagic lesions to nervous disorders, consistent with previous studies using high-virulence CSFV strains, such as the Margarita strain (2, 18).

Interestingly, different replication levels were observed with vPdR-36U and vPdR-5U *in vivo*, despite similar replication kinetics observed in PEDSV.15 cells and in primary porcine macrophages. The PEDSV.15 cells were used to rescue and amplify the viruses for subsequent *in vivo* experiments because we had shown previously that these cells do not select for heparan sulfate-adapted mutants as opposed to other cell lines (19). Infection of piglets with vPdR-5U resulted in significantly higher viral RNA loads in sera and in rectal and nasal swabs than with vPdR-36U. In agreement with previous studies that identified a critical role of the 3′ UTR in replication of pestiviruses and flaviviruses (20–23), our data confirm the relevance of the 3′ UTR in CSFV replication *in vivo* and demonstrate for the first time that a poly-U insertion acquired naturally in the 3′ UTR of CSFV can reduce replication and virulence in young piglets. The RNA secondary structure of the CSFV 3′ UTR was predicted previously, and four consecutive stem-loop (SL) structures were identified (11, 24). Previous studies also revealed that SL-I and the single-stranded intervening sequence (SS) region between SL-I and SL-II are essential for viral replication (20, 24). The predicted RNA secondary structure of the poly-U insertion in the genome of the PdR isolate leads to a long single-stranded intervening sequence between SL-I and SL-II (9). The change in the RNA secondary structure due to this insertion may interfere with the normal function of the 3′ UTR, which influences replication *in vivo* by a yet-unknown mechanism.

Notably, both the vPdR-36U and vPdR-5U viruses generated in the present study were capable of infecting contact piglets by the natural route. For each of the two viruses, the clinical signs, replication rates, and antibody responses were comparable between the inoculated and the respective contact animals, which is supported statistically. Additionally, sequence analysis of the virus recovered from serum at 3 weeks postinfection showed also similar profiles between the inoculated animals and their respective contact groups. Surprisingly however, the 36-uridine sequence of vPdR-36U was not completely stable *in vivo*. Nevertheless, there was no selection of a specific variant (36 or 5 uridines) during natural virus transmission to contact animals.

Type I interferons (IFN), such as IFN-α, induce antiviral and immunomodulatory effects, being a relevant part of the innate immune responses against viruses and hindering viral dissemination (25). For CSFV, transient elevated IFN-α levels are related to disease severity after infection with highly virulent strains (14, 26). In the present study, the vPdR-36U-infected piglets had more consistent and prolonged serum IFN-α levels than the vPdR-5U-infected piglets during the first 2 weeks of infection, suggesting a role for the poly-U sequence in the activation of innate antiviral immune responses. This may be one reason for the lower replication of vPdR-36U versus vPdR-5U in infected piglets, contrasting with the similar replication kinetics of the two viruses in cell culture (Fig. 1), in which CSFV prevents efficiently type I IFN induction by means of N^pro^-mediated IRF3 degradation (27). This is not surprising since functional N^pro^ is present in both vPdR-36U and vPdR-5U. Notably, the highest IFN-α levels overall were found during the first week in some of the vPdR-5U-infected piglets. These were similar to the levels found previously in piglets infected with the highly virulent Margarita strain (18). Interestingly also, the vPdR-36U-infected piglets had serum IFN-α levels that were comparable to those observed previously in piglets infected with the low-virulence PdR strain, considering that this cytokine was detected over a longer period for these animals (7). It should be noted here that the 3′ UTR in *Flaviviridae* has been implicated in the modulation of type I IFN responses and virus replication in different ways. For instance, flaviviruses can inhibit type IFN responses by means of a short noncoding RNA issued from the 3′ UTR (12, 28). Conversely, a long poly-U/UC tract found in the 3′ UTR of hepatitis C virus was shown to activate innate immune responses, including the type I IFN pathway, and control infection through the engagement of RIG-I (13). This points toward a potential role for the long poly-U sequences in the 3′ UTR of CSFV in the activation of the innate immune response, which requires further investigation.

In line with a more sustained innate immune activation by vPdR-36U than by vPdR-5U, the former also induced adaptive immune responses as measured by total and neutralizing antibodies. This contrasted with vPdR-5U, which did not induce any seroconversion during the 4 weeks the piglets survived. The CSFV-specific humoral immune responses were particularly high in animals showing a milder disease and lower viral load. Thus, our results strongly suggest a role for the long poly-U sequence of the PdR isolate in immune activation, control of viral replication, and eventually modulation of disease severity. This is supported by the severe clinical signs observed following vPdR-5U infection, associated with the high replication rate and the incapacity to induce antibody responses. The findings of the present study, i.e., the attenuation of a CSFV strain by the poly-U insertion in the 3′ UTR, provide a potential target for an efficient control of CSFV infection *in vivo* that could be considered in the development of new vaccines against infections with CSFV and other flaviviruses. From a general point of view, our data contribute to understanding of the molecular determinants of CSFV virulence, which supports vaccine development and the implementation of efficient CSF surveillance and control measures.

## MATERIALS AND METHODS

### Cells and viruses.

The porcine kidney cell line PK-15 was obtained from the ATCC (CCL-33) and the SK-6 cell line (29) was provided by M. Pensaert, Faculty of Veterinary Medicine, Ghent, Belgium. The porcine aortic endothelial cell line PEDSV.15 (30) was kindly provided by J. Seebach, University of Geneva, Switzerland. The PK-15 cells were grown in minimum essential medium (MEM) supplemented with 10% pestivirus-free fetal bovine serum (FBS). The SK-6 and PEDSV.15 cells were propagated in Dulbecco’s modified Eagle medium (DMEM) supplemented with sodium pyruvate, nonessential amino acids, 7% horse serum, and 2% porcine serum. Porcine monocyte-derived macrophages were prepared as described previously (31). Briefly, peripheral blood mononuclear cells were isolated from the blood of 6- to 12-month-old specific-pathogen-free Large White pigs from the breeding facility of the Institute of Virology and Immunology (IVI) in Switzerland (in compliance with the Swiss animal protection law, under license number BE131/17, approved by the animal welfare committee of the canton of Bern, Switzerland) using Ficoll-Paque Plus density centrifugation (GE Healthcare). Monocytes were then enriched by positive selection for CD172a with the monoclonal antibody clone 74-22-15A (hybridoma kindly provided by A. Saalmüller, Veterinary University of Vienna, Austria) using a magnetic cell sorting system (MACS) with LS columns (Miltenyi Biotec GmbH). The enriched monocytes were seeded at a density of 5 × 10^5^ cells per milliliter in DMEM without phenol red, supplemented with Glutamax (Life Technologies), 10% pestivirus-free FBS, and recombinant porcine colony-stimulating factor 1 (20 U/ml), and cultured at 39°C with 5% CO_2_ for 72 h for differentiation to macrophages.

For virus amplification, PEDSV.15 cells were infected with 0.1 50% tissue culture infective dose (TCID_50_)/cell in the presence of 2% serum, and the virus was harvested 72 h later. Viral titers were determined by endpoint dilution in PEDSV.15, PK-15, and SK-6 cells using the peroxidase‐linked assay (PLA) (32), and the TCID_50_ per milliliter was calculated using standard statistical methods (33). The CSFV PdR isolate (CSF1058) originated from the Cuban CSF epizootic in 2010 (9). The CSFV reference strain Alfort/187 was kindly provided by the CSFV EU Reference Laboratory (EURL), Hannover, Germany, and was used for virus neutralization assays.

### Assembly of full‑length cDNA clones of the PdR isolate.

The strategy for the construction of the full-length functional cDNA clone of the PdR isolate was based on the backbone vector pACNR1180, described previously (34). The polylinker of the pACNR1180 vector was replaced between SalI and XhoI with a short DNA cassette carrying the SalI and PspXI restriction sites. The complete genome of the PdR isolate was amplified from serum of an infected piglet by reverse transcription (RT) and PCR as previously described (9). Briefly, the 5′ and 3′ halves of the genome were assembled in 2 separate pACNR1180-derived constructs termed pPdR-5′h and pPdR-36U-3′h using large overlapping RT-PCR fragments, according to the sequence information (GenBank accession number KX576461) reported earlier (9). A forward oligonucleotide carrying a SalI restriction endonuclease site for insertion in pACNR1180, a bacteriophage T7 polymerase promoter sequence with the single guanosine of the PdR genome at the transcription start site, and the 5′-terminal 22 nucleotides of PdR served to place the 5′ end of the genome at the proper position in pPdR-5′h for precise viral genome transcription. An SrfI restriction endonuclease site for runoff transcription was placed at the 3′ end of the genome in pPdR-36U-3′h using a reverse oligonucleotide complementary to the 20 last nucleotides of PdR and carrying the SrfI site and a PspXI restriction endonuclease site for insertion into the modified pACNR1180 plasmid. The unique SpeI site with cleavage after nucleotide 8273 in the NS4B gene of PdR served as junction site to assemble the full-length cDNA clone pPdR-36U from the pPdR-5′h and pPdR-36U-3′h constructs. The pPdR-36U plasmid was then modified by deletion of 31 uridines from the poly-U sequence of the 3′ UTR to obtain the standard 5 uridines at this position in a plasmid termed pPdR-5U. To this end, a PCR-based method was applied to generate a fragment encoding 5 uridines instead of the 36 uridines, which was used to replace the BamHI-PspXI cassette in pPdR-36U-3′h, resulting in pPdR-5U-3′h. Plasmid pPdR-5U was then assembled from pPdR-5′h and pPdR-5U-3’h using the SpeI junction site.

### Virus rescue from cDNA.

The viruses vPdR-36U and vPdR-5U were rescued from the respective cDNA clones as described previously (35). Briefly, plasmids pPdR-36U and pPdR-5U were linearized with the SrfI restriction endonuclease and served as templates for runoff RNA transcription with the MEGAscript T7 kit (Invitrogen by Thermo Fisher Scientific) according to the manufacturer’s protocol. The *in vitro*-transcribed RNAs were treated with 1 U of DNase I at 37°C for 15 min and purified on S-400 HR columns (GE Healthcare Life Sciences). For electroporation, PEDSV.15 cells were washed twice with ice-cold phosphate-buffered saline (PBS). A total of 8 × 10^6^ cells in 0.4 ml of PBS were mixed with 1 μg of purified RNA transcript, transferred to a 0.2-cm electroporation cuvette (Bio-Rad), and electroporated immediately with a Gene Pulser electroporation device (Bio-Rad) by applying two pulses at 200 V and 500 μF. The electroporated cells were seeded in 75-cm^2^ flasks and incubated at 37°C and 5% CO_2_. After 65 h, the supernatants were harvested and used to infect fresh PEDSV.15 cells. The specific infectivity of the RNA transcripts was determined with an infectious-center assay on PEDSV.15 cells as described earlier to ensure functionality of the constructs (36). The entire genomes of the rescued viruses were verified by nucleotide sequencing to exclude any accidental mutation. The virus titers were determined in PEDSV.15 and PK-15 cells and on porcine monocyte-derived macrophages as described above.

### Virus replication kinetics in cell culture.

PEDSV.15 cells (150,000/well) and porcine macrophages (500,000/well) were infected with the viruses of interest at a multiplicity of infection (MOI) of 0.02 TCID_50_/cell (based on titers obtained in the homologous cell line) in the corresponding serum-free medium in 24-well plates. After 1 h at 37°C, the inoculum was removed and the cells were washed once with serum-free medium and then incubated in complete culture medium at 37°C. At 3, 6, 9, 12, 18, 24, 36, 48, and 72 h (PEDSV.15 only) after infection, the 24-well plates were frozen at –70°C. After thawing, the supernatant and cells debris were harvested, cleared by centrifugation at 3,000 × *g* for 10 min at 4°C, and then stored at –70°C. The virus titers were determined in PEDSV.15 and SK-6 cells as described above.

### Experimental infection.

Sixty 5-day-old piglets from pestivirus-free sows were housed in two boxes of the biosafety level 3 (BSL3) animal facilities at IRTA-CReSA (Barcelona, Spain). Heating panels and lamps as well as chipped-wood bedding were provided in each box. The animals were fed with commercial pig milk substitute with lyophilized bovine colostrum (Patavie; Celtilait Ltd., Ploudaniel, France) until 14 days of age and subsequently with a conventional piglet starter diet and pellets until the end of the trial (Neopigg, Kwikstart; Cargill, Zaragoza, Spain) (37). The piglets were distributed randomly between the two groups. The piglets of the first experimental group (group 1, *n* = 20, numbered 1 to 20) were inoculated intranasally with 2.5 × 10^4^ TCID_50_ of vPdR-36U (according to the titer in PEDSV.15 cells). At 24 h after infection, 10 contact piglets were added to group 1 (contact group 1, numbered 21 to 30). The piglets of the second experimental group (group 2, *n* = 20, numbered 31 to 50) were inoculated intranasally with 2.5 × 10^4^ TCID_50_ of vPdR-5U. Finally, 10 piglets were added to group 2 as contact animals 24 h after infection (contact group 2, numbered 51 to 60). At 7 dpi, nasal and rectal swabs and serum were collected from 10 randomly selected animals/group from groups 1 and 2. Subsequently, these samples were collected from all animals at 7-day intervals until 5 weeks after infection (i.e., the end of the trial).

The animals were monitored daily by a trained veterinarian in a blinded manner. The clinical status of the animals was scored from 0 to 6 as previously described (38): 0, no signs; 1, mild diarrhea; 2, mild clinical signs; 3, mild to moderate clinical signs; 4, moderate clinical signs; 5, moderate to severe clinical signs; and 6, death. For ethical reasons, the animals were euthanized when the clinical score reached 5, when exhibiting a fall of the hindquarters, when there was inability to drink or feed, when prostration occurred, or when exhibiting moderate nervous disorders. The procedure for the euthanasia of the animals was based on an accepted method included in European Directive 2010/63/EU, using an overdose of 60 to 100 mg of pentobarbital per kilogram of body weight, administered via the vena cava cranialis. The experiment was approved by the Ethics Committee from the Generalitat of Catalonia, according to Spanish and European regulations.

### Detection of CSFV RNA.

RNA was extracted using the MagAttract 96 *cador* pathogen kit (Qiagen), following the manufacturer’s instructions. In all cases, extraction was performed from an initial sample volume of 200 μl to obtain a final volume of 100 μl of RNA, which was stored at –80°C. The presence of CSFV RNA in the serum and in nasal and rectal swabs was analyzed by RT-qPCR (39). *C_T_* values equal to or lower than 42 were considered positive. Samples in which fluorescence was undetectable were considered negative. As described previously, *C_T_* values from 10 to 22 were considered high, from 23 to 28 moderate, and between 29 and 42 low RNA viral loads (40).

### Assessment of the humoral immune responses by ELISA and virus neutralization test.

E2-specific antibodies after infection were detected by ELISA using a commercial CSFV antibody test (IDEXX Laboratories, Liebfeld, Switzerland) according to the manufacturer’s recommendations. The samples were considered positive when the blocking percentage was ≥40%. In order to evaluate the neutralizing antibody response after infection, serum samples were also tested by neutralization peroxidase‐linked assay (NPLA) (41) against the Alfort/187 strain. The titers were expressed as the reciprocal dilution of serum that neutralized 100 TCID_50_ in the 50% of the culture replicates.

### ELISA for IFN-α detection in serum samples.

An in-house ELISA was performed in order to quantify IFN-α levels in sera at 8 and 15 dpi as previously described (42, 43). Anti-IFN-α monoclonal antibodies (K9 and K17) were used for detection and serial dilutions of IFN-α recombinant protein (PBL Biomedical Laboratories, Piscataway, NJ) was employed as a standard. A regression curve based on the optical densities of the cytokine standard used in the test was used to determine the cytokine concentrations (units/milliliter) in sera.

### CSFV rescue in serum samples from infected piglets.

Serum samples from all animals of the two experimental groups were subjected to virus isolation at 3 weeks postinfection. To this end, PK-15 cells were seeded in a 96-well plate and incubated at 37°C and 5% CO_2_. After 24 h, 100 μl of 10- and 100-fold dilutions of each sample were added. After 72 h of incubation, the virus was detected by PLA test (32). In addition, the virus titers were determined as described above.

### Analysis of the 3′ UTR nucleotide sequence in serum from infected piglets.

The following two primers were designed for the specific detection of the 3′ UTR sequence: forward primer (12023 to 12049), 5′-AAGGAGGCTGAGAGTCATGATGATGAC-3′, and reverse primer (12304 to 12329), 5′-GGGCCGTTAGGAAATTACCTTAGTCC-3′. RNAs from serum samples from all animals of the two experimental groups were tested at 3 weeks postinfection. The RT-PCRs, consisting of 30 min at 50°C (reverse transcription) and 10 min at 95°C (inactivation of the reverse transcriptase and activation of the *Taq* polymerase) followed by 35 cycles of 30 s at 94°C, 30 s at 54°C, and 45 s at 72°C, were performed using a one-step RT-PCR kit (Qiagen) in a final volume of 25 μl. The amplification products were analyzed by electrophoresis on a 2% agarose gel and were purified with a GeneJET gel extraction and DNA cleanup microkit (Thermo Fisher Scientific). The sequencing reactions were performed using the BigDye terminator cycling technique and an ABI 3130xl genetic analyzer. The sequences were assembled using the Contig BioEdit software (44).

### Statistical analysis.

NCSS software (Hintze. J 2004 NCSS and PASS Number Cruncher Statistical Systems. Kaysville, UT) served for statistical analyses using “piglet” as the experimental unit. The nonparametric Kruskal-Wallis test was used to compare the clinical scores and the virological (viral loads in serum, rectal swabs, and nasal swabs) and immunological (IFN-α levels) parameters among the four experimental groups. The significance level (α) was set at a *P* value of ≤0.05, whereas statistical tendencies were reported when *P* values were ≤0.10.

## Supplementary Material

Supplemental file 1
